# Supplementary material for: GRAPE: genomic relatedness detection pipeline
Source: F1000Res. 2023 Apr 13;11:589. Originally published 2022 May 30. [Version 2] doi: 10.12688/f1000research.111658.2 (PMC10182380; doi:10.12688/f1000research.111658.2)
Supplement: Peer review history

# Peer review: Reviewer response for version 2

**DOI:** 10.5256/f1000research.145655.r169523

**Published:** 2023-05-12

**Authors:** Ying Zhou

**Affiliations:** 1 Public Health Sciences Division, Fred Hutchinson Cancer Research Center, Seattle, WA, USA

I don't have further comments since the authors have well addressed my concerns.

Are the conclusions about the tool and its performance adequately supported by the findings presented in the article?

Yes

Is the rationale for developing the new software tool clearly explained?

Yes

Is the description of the software tool technically sound?

Yes

Are sufficient details of the code, methods and analysis (if applicable) provided to allow replication of the software development and its use by others?

Yes

Is sufficient information provided to allow interpretation of the expected output datasets and any results generated using the tool?

Yes

Reviewer Expertise:

population genetics, computational genetics

I confirm that I have read this submission and believe that I have an appropriate level of expertise to confirm that it is of an acceptable scientific standard.

# Peer review: Reviewer response for version 2

**DOI:** 10.5256/f1000research.145655.r169524

**Published:** 2023-05-04

**Authors:** Ardalan Naseri

**Affiliations:** 1 School of Biomedical Informatics, University of Texas Health Science Center at Houston, Houston, TX, USA

The authors have addressed all of my comments. I do not have any more concerns.

Are the conclusions about the tool and its performance adequately supported by the findings presented in the article?

Yes

Is the rationale for developing the new software tool clearly explained?

Yes

Is the description of the software tool technically sound?

Yes

Are sufficient details of the code, methods and analysis (if applicable) provided to allow replication of the software development and its use by others?

Yes

Is sufficient information provided to allow interpretation of the expected output datasets and any results generated using the tool?

Yes

Reviewer Expertise:

Computational Biology, Bioinformatics, Population Genetics

I confirm that I have read this submission and believe that I have an appropriate level of expertise to confirm that it is of an acceptable scientific standard.

# Peer review: Reviewer response for version 1

**DOI:** 10.5256/f1000research.123378.r143937

**Published:** 2022-08-03

**Authors:** Ardalan Naseri

**Affiliations:** 1 School of Biomedical Informatics, University of Texas Health Science Center at Houston, Houston, TX, USA

Medvedev et al. have developed a user-friendly software/pipeline for relatedness inferences from genetic data. The pipeline includes data preprocessing, identity-by-descent (IBD) segment detection, and relationship inferences. The pipeline has incorporated several software inside a Docker that enables a very convenient solution for users aiming to infer relationships from genetic data. 

 The authors have documented the performance of the pipeline regarding the accuracy and run time using both simulated and real-world data. There are a few suggestions/comments that could improve this work:

1. The memory usage of the pipeline has not been mentioned in the article. It would be useful to provide more information about the required memory usage.
2. Current biobank-scale data contain hundreds of thousands of individuals. The run time for 100k individuals was reported as 22 hours using 8 CPU cores (for IBIS + ERSA). The user may want to know if they can run the pipeline in large panels within a reasonable time. The run time of the pipeline using IBD detection (with GERMLINE) was also not included. More recent IBD detection tools (e.g. hapIBD, RaPID and iLASH) can handle very large cohorts efficiently. Incorporating other IBD detection tools might also improve the efficiency of the pipeline regarding the run time and/or memory usage.
3. Robustness against genotyping errors, different marker densities, and different populations have not been studied well. The authors have pointed out the limitation with a small isolated population though. 

Are the conclusions about the tool and its performance adequately supported by the findings presented in the article?

Yes

Is the rationale for developing the new software tool clearly explained?

Yes

Is the description of the software tool technically sound?

Yes

Are sufficient details of the code, methods and analysis (if applicable) provided to allow replication of the software development and its use by others?

Yes

Is sufficient information provided to allow interpretation of the expected output datasets and any results generated using the tool?

Yes

Reviewer Expertise:

Computational Biology, Bioinformatics, Population Genetics

I confirm that I have read this submission and believe that I have an appropriate level of expertise to confirm that it is of an acceptable scientific standard, however I have significant reservations, as outlined above.

# Response

**Published:** 2023-03-15

**Authors:** Aleksandr Medvedev

Medvedev et al. have developed a user-friendly software/pipeline for relatedness inferences from genetic data. The pipeline includes data preprocessing, identity-by-descent (IBD) segment detection, and relationship inferences. The pipeline has incorporated several software inside a Docker that enables a very convenient solution for users aiming to infer relationships from genetic data.

 The authors have documented the performance of the pipeline regarding the accuracy and run time using both simulated and real-world data. There are a few suggestions/comments that could improve this work:

1. The **memory usage** of the pipeline has not been mentioned in the article. It would be useful to provide more information about the required memory usage.
   
  1. **Answer:** We implemented several batch preprocessing and postprocessing routines to ensure that for the dataset of every size memory usage will not be more than 16GB RAM.
2. Current **biobank-scale data** contain hundreds of thousands of individuals. The run time for 100k individuals was reported as 22 hours using 8 CPU cores (for IBIS + ERSA). The user may want to know if they can run the pipeline in **large panels** within a reasonable time. The run time of the pipeline using IBD detection (with **GERMLINE**) was also **not included**. More recent IBD detection tools (e.g. **hapIBD, RaPID and iLASH)** can handle very large cohorts efficiently. Incorporating other IBD detection tools might also improve the efficiency of the pipeline regarding the run time and/or memory usage.
   
  1. **Answer:** Based on Figure 3 and the fact that IBIS and ERSA algorithms has O(n^2) complexity, when n is the number of samples, we can estimate that running time on n~=500k will be ~20-25 days. This is comparable with time required for phasing a dataset of this size. We are planning to investigate accuracy and performance on hapIBD, RaPID and iLASH for phased datasets, but unique advantage of IBIS-based workflows is that it does not require phasing. We chose GERMLINE as the well-known method with an appropriate license. iLASH does not have an MIT or GPL-like license.
  2. **Answer: **In the new article version we added beta RaPID support and compared it with IBIS and GERMLINE. RaPID-based workflow took 45 min to run on 100K dataset and 6 hours to run on 500K dataset. But the dataset was already phased.
3. **Robustness against genotyping errors, different marker densities, and different populations** have not been studied well. The authors have pointed out the limitation with a small isolated population though.
   
  1. **Answer:** IBIS-based workflows detect relatively big IBD segments (more than 5-7 cM). We hope that it will provide some robustness to genotyping error. We also provide a —remove imputation option in the preprocessing workflow, because we found out that some imputed regions produce many false-positive IBD matches.
  2. **Answer: **IBIS and RaPID-based workflows should not detect many false-positive IBD segments in the low-density regions, because they constrain both minimum number of SNPs in an IBD match and minimum cM length. However, low-density regions could produce many false-negatives.

# Peer review: Reviewer response for version 1

**DOI:** 10.5256/f1000research.123378.r139363

**Published:** 2022-07-12

**Authors:** Ying Zhou

**Affiliations:** 1 Public Health Sciences Division, Fred Hutchinson Cancer Research Center, Seattle, WA, USA

Medvedev et al. presented a user-friendly pipeline for detecting both the distant and the close relatedness. The design is straightforward, and this pipeline includes different inference strategies that can deal with both phased and unphased data, and it can be run inside a Docker container, which makes it very applicable in real analysis. It is also appreciable that authors devoted lots of times in building this pipeline. I have only three comments:

1. I may understand why to use the King software for close relatedness inference while using IBD based method for distant relatedness inference. It is because large IBD cutoff for example 7cM could lead to loss of IBD segments and bias the kinship estimation. However, when the target population is structured or admixed, the King software may not work as well as IBD based inference.
2. What should I do if I need to run analysis on a large sample size, for example, UK biobank (n~=500k)? The largest test data in this work is 100k, while some other methods declare they can deal with kinship estimation on much larger sample sizes, see ref ^1^.
3. If I want to find out all potential relatives of one target sample in a reference database, with this pipeline, I need to merge the data first and then detect all relatedness. This is not efficient. An alternative solution is to use query based IBD detection, see ref ^2^.

Are the conclusions about the tool and its performance adequately supported by the findings presented in the article?

Yes

Is the rationale for developing the new software tool clearly explained?

Yes

Is the description of the software tool technically sound?

Yes

Are sufficient details of the code, methods and analysis (if applicable) provided to allow replication of the software development and its use by others?

Yes

Is sufficient information provided to allow interpretation of the expected output datasets and any results generated using the tool?

Yes

Reviewer Expertise:

population genetics, computational genetics

I confirm that I have read this submission and believe that I have an appropriate level of expertise to confirm that it is of an acceptable scientific standard, however I have significant reservations, as outlined above.

## References

[1] : IBDkin: fast estimation of kinship coefficients from identity by descent segments. *Bioinformatics* .2020;36(16) : 10.1093/bioinformatics/btaa569 4519-4520 10.1093/bioinformatics/btaa569 32844204PMC7750976

[2] : Efficient haplotype matching between a query and a panel for genealogical search. *Bioinformatics* .2019;35(14) : 10.1093/bioinformatics/btz347 i233-i241 10.1093/bioinformatics/btz347 PMC661285731510689

# Response

**Published:** 2023-03-15

**Authors:** Aleksandr Medvedev

1. I may understand why to use the KING software for close relatedness inference while using IBD based method for distant relatedness inference. It is because a large IBD cutoff for example 7cM could lead to a loss of IBD segments and bias the kinship estimation. However, **when the target population is structured or admixed**, the **KING** software **may not work** as well as **IBD-based inference**. [paper updated]
   
  1. **Answer:** For the close relatedness inference we use the KING ibdseg option by default, which also uses IBD segments for the degree estimation. Also, we calculate the kinship coefficient using the legacy KING kinship option and also report kinship-based degrees in our output file. We changed the description of the usage of KING in our paper.
2. What should I do if I need to run the analysis on a large sample size, for example, UK biobank (n~= **500k**)? The largest test data in this work is 100k, while some other methods declare they can deal with kinship estimation on much larger sample sizes, see https://academic.oup.com/bioinformatics/article/36/16/4519/5858978.
  1. **Answer:** Based on Figure 3 and the fact that the IBIS and ERSA algorithms have O(n^2) complexity when n is the number of samples, we can estimate that running time on n~=500k will be ~20-25 days on the system specified in our article. This is comparable with time required for phasing a dataset of this size. We looked into IBDKin and we are willing to evaluate it in our future GRAPE version as a King substitution. However, as far as we understand, it does not infer distant degrees yet.
  2. **Answer: **In the new article version we added beta RaPID support and compared it with IBIS and GERMLINE. RaPID-based workflow took 45 min to run on the 100K dataset and 6 hours to run on the 500K dataset. But the dataset was already phased.
3. If I want to find out all potential relatives of **one target sample** **in a reference database**, with this pipeline, I need to merge the data first and then detect all relatedness. This is not efficient. An alternative solution is to use **query-based IBD detection**, see https://academic.oup.com/bioinformatics/article/35/14/i233/5529240.
  1. **Answer:** We plan to implement a query-based IBD detection for IBIS in one of the future GRAPE releases. We checked the paper about PBWT queries and it appears that it works only with haplotypes and not genotypes, i.e. phased data. However, we plan to test RaPID and hapIBD on phased data as a GERMLINE replacement and possibly implement query-based IBD detection for them.
